# Hylin-a1: A Host Defense Peptide with Antibacterial Potential against *Staphylococcus aureus* Multi-Resistant Strains

**DOI:** 10.3390/ph16040509

**Published:** 2023-03-29

**Authors:** Annalisa Chianese, Carla Zannella, Francesco Foglia, Bianca Maria Nastri, Alessandra Monti, Nunzianna Doti, Gianluigi Franci, Anna De Filippis, Massimiliano Galdiero

**Affiliations:** 1 Department of Experimental Medicine, University of Campania “Luigi Vanvitelli”, 80138 Naples, Italy; 2 Institute of Biostructures and Bioimaging (IBB), National Research Council (CNR), 80131 Naples, Italy; 3 Centro Interuniversitario di Ricerca sui Peptidi Bioattivi (CIRPEB), 80134 Naples, Italy; 4 Department of Medicine, Surgery and Dentistry “Scuola Medica Salernitana”, University of Salerno, 84081 Baronissi, Italy

**Keywords:** AMP, *Staphylococcus aureus*, multi-drug resistance, AMPs, peptides, antibacterial, inflammation

## Abstract

In recent years, the resistance of pathogenic microorganisms to common antimicrobial agents has raised to a severe public health problem. The moderate and wise use of antimicrobials and the prevention of infections are the most effective strategies for decreasing the spread and development of resistance. Therefore, the World Health Organization (WHO) has intensified the search for new drugs to fight emerging pathogens. Antimicrobial peptides (AMPs), also known as host defense peptides (HDPs), play a crucial role in innate immunity, representing one of the first line of defense against microbial attacks. In this study, we evaluated the antibacterial activity of the AMP named Hylin-a1 (derived from the skin of the frog *Heleioporus albopunctatus*) against *Staphylococcus aureus* strains. *S. aureus* represents a commensal bacterium but also the principal causative agent of several human infections, including bacteremia, endocarditis, skin and device-related infections. Hylin-a1 toxicity was evaluated on human keratinocytes; once the non-cytotoxic concentration range was determined, the minimum inhibitory concentration (MIC) and minimum bactericidal concentration (MBC) were analyzed, and time-killing assays were performed to verify the bacteriostatic and/or bactericidal activity of the peptide. We found that Hylin-a1 exerted a bacteriostatic action against most of the tested strains, with 90% inhibition at the concentration of 6.25 μM. Noteworthy, the peptide at a very low concentration (~3 μM) significantly blocked the growth of β-lactam- and methicillin-resistant *S. aureus*. The levels of interleukin (IL)-1β, IL-6 and IL-8 were quantified through a molecular assay, indicating that the peptide was able also to regulate the inflammatory response following bacterial infection. The effect of Hylin-a1 on *S. aureus* cell morphology was also evaluated. Altogether, these results indicate the high therapeutic potential of Hylin-a1 against a wide variety of clinical manifestations caused by *S. aureus*.

## 1. Introduction

To date, the resistance of most human pathogenic bacterial strains to antibiotics has rapidly increased. As stated by the World Health Organization (WHO), the use and abuse of broad-spectrum antimicrobial drugs have led to the emergence of pathogens that are multi-resistant to conventional antibiotics and do not respond to the common therapies, representing a severe challenge to the global public health [1]. From 2019, it is estimated that most of the deaths per year in the European Union (approximately 25,000) and worldwide (about 1270,000) have been caused by pathogens resistant to antibiotic therapies [2]. Currently, *Staphylococcus aureus* (*S. aureus*) represents one of the most critical and problematic bacterial pathogens [2,3].

*S. aureus* is a catalase-positive Gram-positive bacterium belonging to the *Micrococcaceae* family [4]. It represents one of the most dangerous and versatile pathogens for humans, responsible for various diseases with clinical outcomes ranging from mild to severe. 

*S. aureus* colonizes different hosts districts, causing superficial skin and soft tissue infections, respiratory tract diseases, sepsis and nosocomial bacteremia [5,6,7]. In 1928, with the discovery of penicillin, many bacterial infections caused by *S. aureus* became treatable. However, the first resistant strains producing penicillinase, an enzyme capable of hydrolyzing penicillin and inactivating it, were identified in a short time [8,9,10]. To overcome this obstacle, many pharmaceutical companies started to market methicillin in 1960, a penicillinase-resistant beta-lactam antibiotic, to treat infections caused by *S. aureus* [11]. However, as early as 1961, the first phenomenon of methicillin resistance in strains of *S. aureus* occurred, due to the production of an extra penicillin-binding protein (PBP2a) with reduced affinity for beta-lactam [12]. This event represented a severe problem, since methicillin-resistant *S. aureus* (MRSA) strains became resistant to a broad spectrum of antibiotics (i.e., penicillins, cephalosporins, carbapenems, macrolides) [13].

Therefore, effective infection control measures and the development of new classes of antimicrobial agents require rapid and extensive efforts [14,15,16,17]. In this framework, particular interest has been aroused by the discovery of antimicrobial peptides (AMPs) which exert a well-known lethal activity on many invading microorganisms [18,19,20]. 

To date, AMPs are promising candidates for a new generation of anti-infective agents [21,22]. In detail, they have showed a remarkable efficacy against multi-drug resistant bacteria with a low propensity to induce resistance compared to other antimicrobial drugs [23]. Their biological activity is ascribed to the direct killing of pathogenic microbes or indirectly to the modulation of the innate immune system [19,24]. AMPs interact with the bacterial cell membrane or with intracellular targets (such as the synthesis, modulation, and folding of proteins) through two main mechanisms [25]: they can cross the cell membrane and interfere with cellular components [26], or disrupt the cell membrane with a detergent action [19,27,28]. Peptides with antimicrobial activity have been identified in bacteria, insects and amphibians [16,29]. For example, temporins represent one of the most prominent families of peptides deriving from the skin secretions of the common frog *Rana temporaria* [30,31,32]. They are known to be particularly active against Gram-positive bacteria, with minimum inhibitory concentrations (MIC) between 2.5 and 20 µM [31,33]. We have also shown that temporin L is active against Gram-negative bacteria and fungi, and furthermore, that changes in the primary sequence of the peptide drastically improve the toxicity profile of the peptide [34].

In the present study, we analyzed the antibacterial effect of the peptide Hylin-a1 by focusing on the inhibition of infections by *S. aureus* and a set of multi-resistant clinical isolates. Castro MS. et al. isolated the 18-mer peptide for the first time from the skin secretion of the frog *Hypsiboas albopunctatus* in 2009 [35]. The peptide exhibited a broad range of activity mainly against Gram-positive bacteria, but also against fungi, probably due to its amphipathic a-helical structure. The peptide has been then modified by introducing different charges at the N-terminal end in order to improve its antimicrobial effect [36], and the peptide carrying a positive charge showed an increased activity against bacteria and fungi compared to the non-mutated peptide. Recently, Hylin-a1 and several analogs were analyzed for their ability to interfere with *Acinetobacter baumannii* (*A. baumannii*) infection, and some of the peptides showed a potent effect also against clinical isolates [37], inducing the disruption of the integrity of the bacterial membrane. These findings, together with our data, suggest that Hylin-a1 and derived peptides can be used as effective treatment options against infections caused by multi-drug resistant bacteria.

## 2. Results and Discussion

### 2.1. Hylin-a1 Cytotoxicity Evaluation

**Figure 1:**
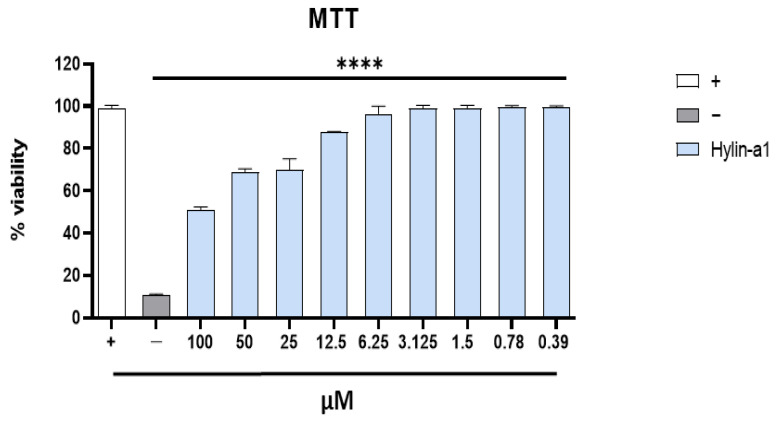
Evaluation of the toxicity of Hylin-a1 in HaCaT cells. Peptide toxicity was evaluated by the MTT assay after 20 h. Untreated cells were used as a positive control (+), while DMSO-treated cells were used as a negative control (−). **** *p* < 0.0001.

The effect of Hylin-a1 on cell viability was examined via a specific colorimetric assay, named 3-(4,5-dimethylthiazol-2-yl)-2,5-diphenyl-2H-tetrazolium bromide (MTT). Human keratinocytes (HaCaT) were incubated with different concentrations of the peptides (ranging from 100 to 0.39 µM) for 20 h. Next, the cytotoxic activity was evaluated by monitoring cell viability and expressed as a percentage of viability relative to the viability of untreated cells (+) (Figure 1). Non-linear regression analysis indicated that the 50% cytotoxic concentration (CC_50_) of Hylin-a1 was 100 μM.

### 2.2. Hylin-a1 Antibacterial Activity

#### 2.2.1. Minimum Inhibitory Concentration (MIC) and Minimum Bactericidal Concentration (MBC)

**Figure 2:**
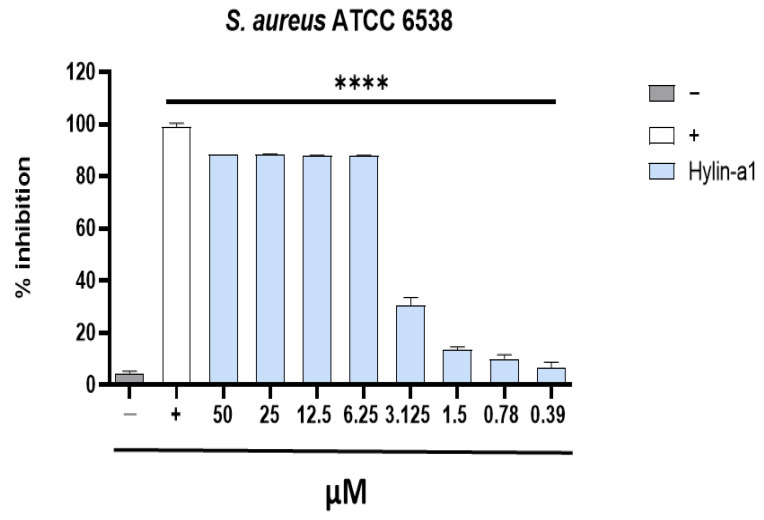
Hylin-a1 antibacterial activity against *S. aureus* ATCC. A microdilution assay was performed in order to evaluate the activity against *S. aureus* ATCC. As a positive control (+) vancomycin (6.9 μM) was used, while bacterial medium represented the negative control (−). **** *p* < 0.0001.

The antimicrobial activity of Hylin-a1 was tested against *S. aureus* ATCC 6538 and clinical isolates. The peptide resulted in inhibiting all the tested strains: the MIC value was 6.25 µM for the ATCC strain of *S. aureus* and in the range from 6.25 to 3.125 µM for the clinical isolates (Figure 2).

In detail, the MIC value was 6.25 µM for the ATCC strain, very similar to that observed in literature [35]. 

It is known that *S. aureus* produces beta-lactamase enzymes capable of inactivating beta-lactam antibiotics [38]. MRSA strains show a reduced affinity for these antibiotics due to the presence of the penicillin-binding protein PBP2a, which prevents the blocking of cell wall synthesis [39]. The bacterial strains analyzed in our study are resistant to antibacterial drugs belonging to different classes. Some interfere with the synthesis of the bacterial cell wall by inhibiting its growth (beta-lactams, MRSA), while others act at the intracellular level either by inhibiting protein synthesis (*macrolide-resistant S. aureus*) or by acting on DNA replication (*quinolone-resistant S. aureus*) [40,41].

**Figure 3:**
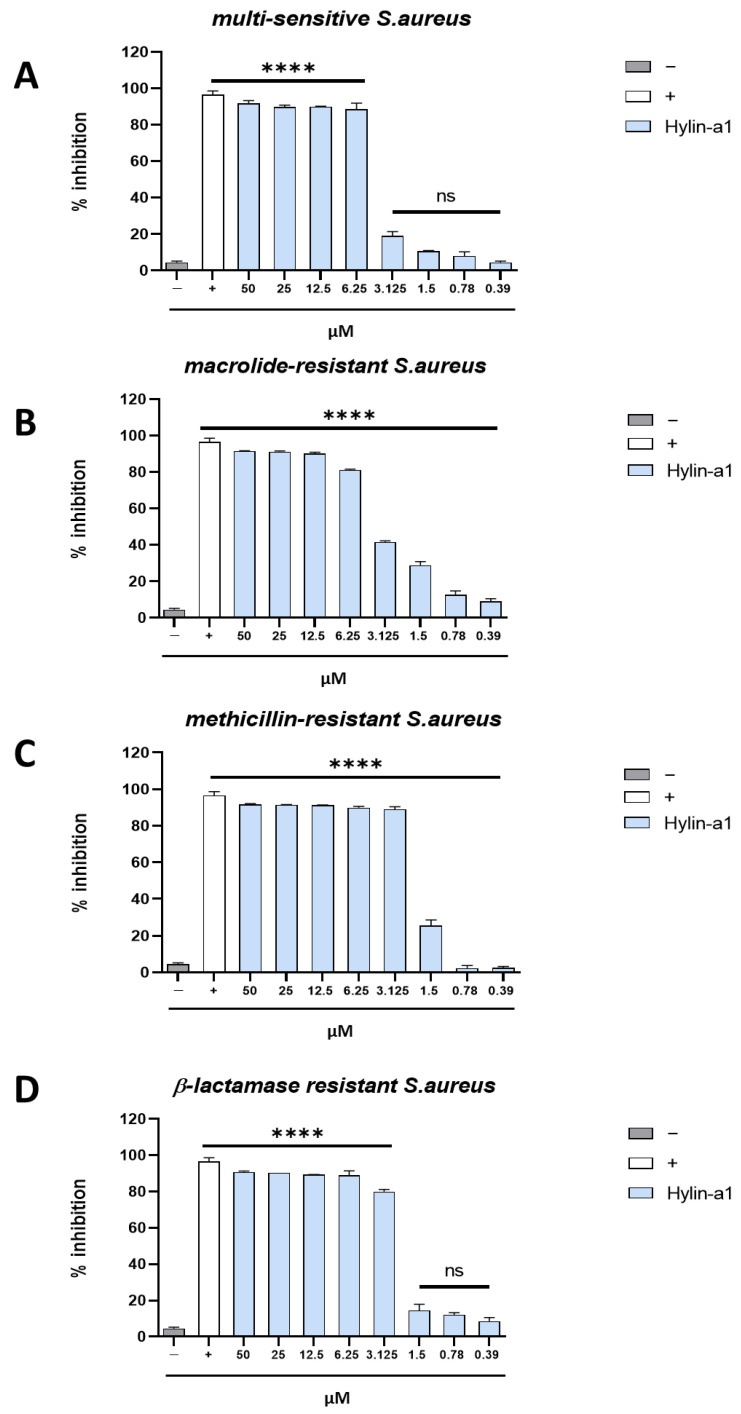

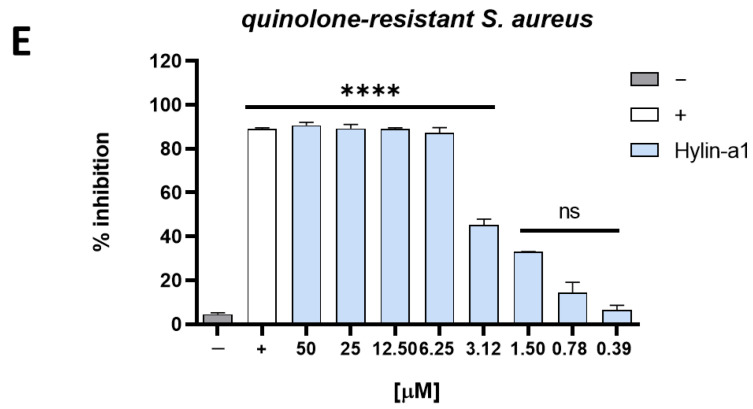
Antibacterial activity against *S. aureus* strains. A microdilution assay was performed to evaluate the activity against multi-sensitive (**A**), macrolide-resistant (**B**), methicillin-resistant (**C**), β-lactamase resistant (**D**) and quinolone-resistant *S. aureus* (**E**). The peptide was tested at different concentrations ranging from 50 to 0.39 µM. As a positive control (+), vancomycin was used (6.9 μM), while bacterial medium represented the negative control (−). **** *p* < 0.0001; ns: non-significant.

The antibacterial effect of Hylin-a1 was therefore also tested against a multi-sensitive strain and four types of resistant *S. aureus* clinical strains: macrolide-resistant, methicillin-resistant (MRSA), β-lactamase resistant, and quinolone-resistant *S. aureus* (Figure 3). 

Hylin-a1 strongly inhibited macrolide-resistant and quinolone resistant strains very similarly to what observed for the multi-sensitive strain (Figure 3A,B,E), retaining the same MIC (6.25 µM) previously observed with the ATCC strain (Figure 2). 

Instead, against the β-lactamase-resistant and MRSA strains, the antimicrobial effect of the peptide was improved, with a MIC value of 3.125 μM (Figure 3C,D).

We observed a different inhibitory effect of Hylin-a1 that was strongly related to the bacterial strains used in our experimental procedure. Probably, the peptide was able to act on the bacterial membrane by interfering with the cell wall rather than with intracellular targets. The MBC values were analyzed to understand if the peptide also could exhibit a bactericidal action. The MBC corresponds to the lowest peptide concentration capable of causing the elimination of more than 99.9% of bacteria [42]. After the MIC determination of Hylin-a1, 50 μL of medium from all the wells showing no visible bacterial growth was seeded on BHI agar plates and incubated O/N at 37 °C. Then, after the incubation, we evaluated the presence or absence of bacteria on the agar plates. If the peptide exerted a bactericidal action, no bacterial growth was observed on the plates [43]. The results are summarized in Table 1 and showed that Hylin-a1 did not exert a bactericidal activity against all bacterial strains tested except for the ATCC derived strain.

#### 2.2.2. Time-Killing Assay

**Figure 4:**
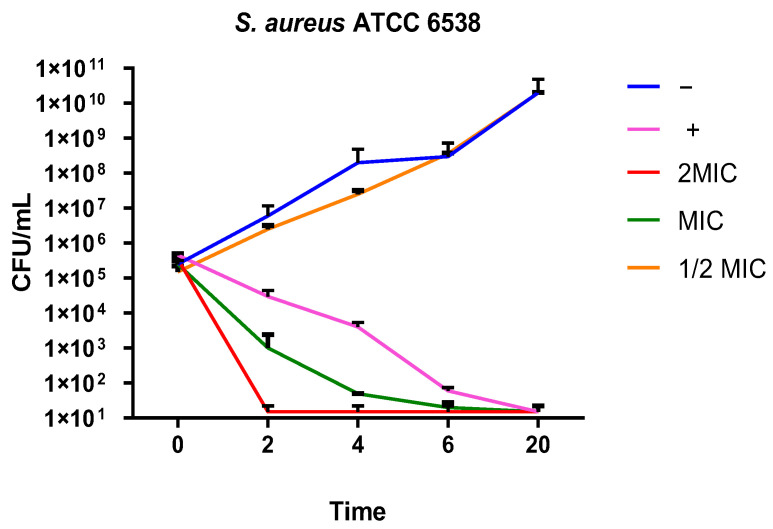
Time-killing assay. Killing kinetics of Hylin-a1 against *S. aureus* strains. (−): bacteria treated with the solvent used to dissolve the drug; (+): bacteria treated with vancomycin (6.9 µM); (2MIC): bacteria treated with Hylin-a1 (12.5 µM); (MIC): bacteria treated with Hylin-a1 (6.25 µM); (½MIC): bacteria treated with Hylin-a1 (3.125 µM).

Our results were also confirmed by monitoring bacterial growth over time. A time-killing assay showed a similar trend of Hylin-a1 action against *S. aureus* ATCC (Figure 4) and all the clinical isolates. As shown in Figure 4, the growth curve of the untreated bacteria increased exponentially over time. 

Similarly, the bacteria treated with Hylin-a1 at 3.25 μM (½ MIC) did not show a significant change in their growth. A significant result was observed with 6.25 and 12.5 μM Hylin-a1 (MIC and 2MIC values), with no rise in the bacterial growth between 0 and 20 h compared to the initial bacterial density, indicating a bacteriostatic action of the tested AMP (Figure 4). Interestingly, the peptide showed a stronger bactericidal action compared to vancomycin, used as a positive control (Figure 4). It caused bacterial death after 2 and 4 h at the MIC and 2MIC values, respectively.

#### 2.2.3. Evaluation of the Invasion Capability

The most important steps in host cell infection are the attachment and subsequent invasion of the bacteria. To understand whether Hylin-a1 protects cells against *S. aureus* infection, we evaluated the capability of the peptide to inhibit bacterial invasion. In the invasion assay, HaCaT cells were treated with different concentrations of Hylin-a1 and then infected with *S. aureus*.

**Figure 5:**
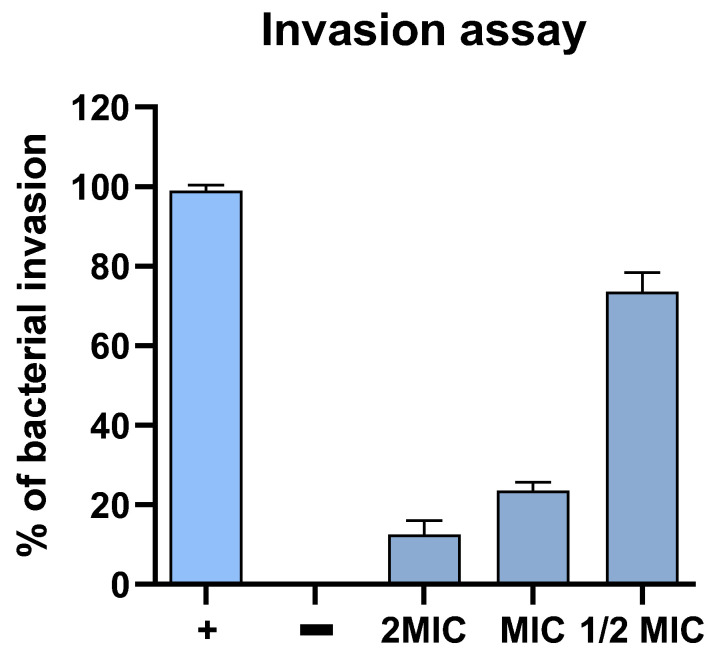
Invasion assay of *S. aureus* in HaCaT cells. HaCaT were treated with different concentrations of Hylin-a1 and then infected with *S. aureus*. (+) Cells treated with *S. aureus* bacteria; (−) cells not treated; (2MIC): cells treated with Hylin-a1 (12.5µM); (MIC): cells treated with Hylin-a1 (6.25 µM); (½MIC): cells treated with Hylin-a1 (3.125 µM). Data represent mean values + standard errors of the means of duplicate experiments.

As reported in Figure 5, *S. aureus* invasion was significantly modulated by Hylin-a1. Indeed, the peptide at 12.5 µM (2MIC) and 6.25 µM (MIC) concentrations effectively reduced bacterial invasion. 

However, the lower concentration of 3.25 µM (½MIC) had no effect on bacterial invasion. These results indicated that Hylin-a1 played a protective role against *S. aureus* infection

### 2.3. Antibiofilm Activity

Biofilms show a higher resistance to antibiotics than single bacteria; therefore, it is necessary to identify new agents capable to inhibit or reduce biofilm formation. One of the initial stages of biofilm formation and development is cell attachment [44,45].

The inhibitory capacity of Hylin-a1 was tested by performing four different assays evaluating the peptide’s ability to inhibit cell attachment and degrade and inhibit biofilm formation.

**Figure 6:**
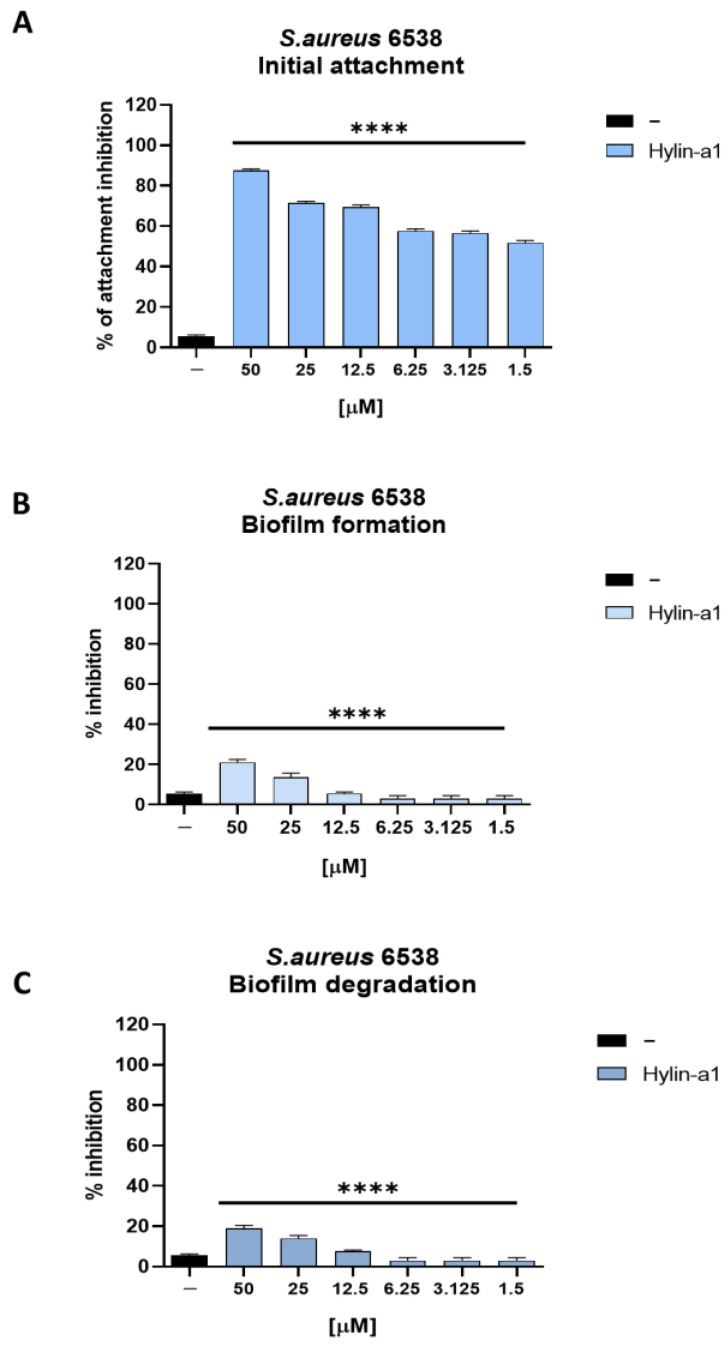
The effect of Hylin-a1 on the biofilm of *S. aureus*. (**A**) Initial cell attachment for biofilm formation; (**B**) biofilm formation; (**C**) biofilm degradation. **** *p* < 0.0001.

To evaluate the inhibitory activity of the peptide on the initial cell attachment required for biofilm formation, a high-density bacterial culture of *S. aureus* was incubated with different concentrations of the peptide for 1 h, and the effect was evaluated by the MTT assay (as described in the Materials and Methods section). Our results proved to be very promising and showed a 50% inhibition at the lowest concentration tested (1.5 µM) (Figure 6a). However, the peptide did not show any inhibitory effect in biofilm formation and degradation assays (Figure 6b,c).

### 2.4. FACScan Analysis

**Figure 7:**
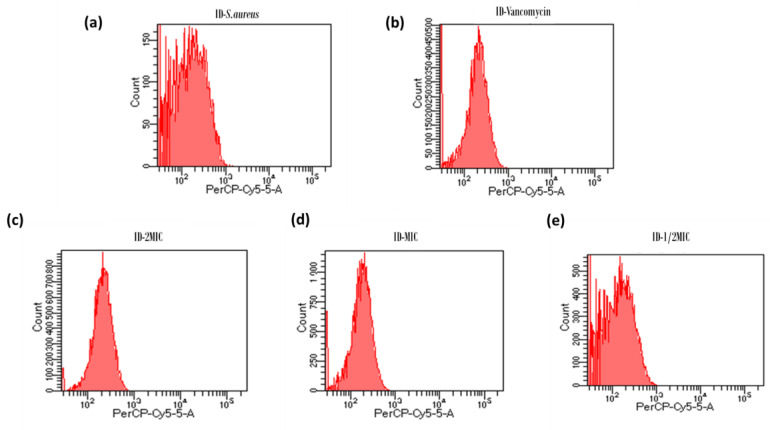
Flow cytometric analysis. The DNA-intercalating dye propidium iodide (PI) was used to evaluate the cell membrane integrity via flow cytometry. The fluorescence intensity was monitored after treating with Hylin-a1. Flow cytometry was performed using a FACScan instrument. (**a**) Untreated bacteria, control; (**b**) bacteria treated with vancomycin (6.9 μM); (**c**) bacteria treated with Hylin-a1 at 2MIC concentration; (**d**) bacteria treated with Hylin-a1 at MIC concentration; (**e**) bacteria treated with Hylin-a1 at ½MIC concentration.

Cell membrane integrity was assessed using propidium iodide (PI), a nucleic acid-intercalating dye used as an indicator of cell death. Fluorescence intensity was evaluated by flow cytometry analysis, which indicated that the peptide promoted the influx of PI into the cells. In Figure 7 it is possible to observe how the median fluorescence signal changed in the various conditions, showing a reduced value (61 and 64) for untreated bacteria and for bacteria treated with the ½MIC concentration of Hylin-a1, confirming membrane integrity (Table 2). 

However, higher concentrations of the peptide resulted in a reduction of the membrane integrity, showing a median fluorescence signal comparable to that measured for bacteria treated with vancomycin (positive control) (Figure 7). These results are consistent with those of the antibacterial studies, indicating that Hylin-a1 had strong binding and penetrating activities with respect to *S. aureus* cell membrane. 

### 2.5. SEM Analysis

Surface roughening and cell membrane disruption of *S. aureus* treated with the antimicrobial peptide Hylin-a1 were also observed and confirmed by SEM.

**Figure 8:**
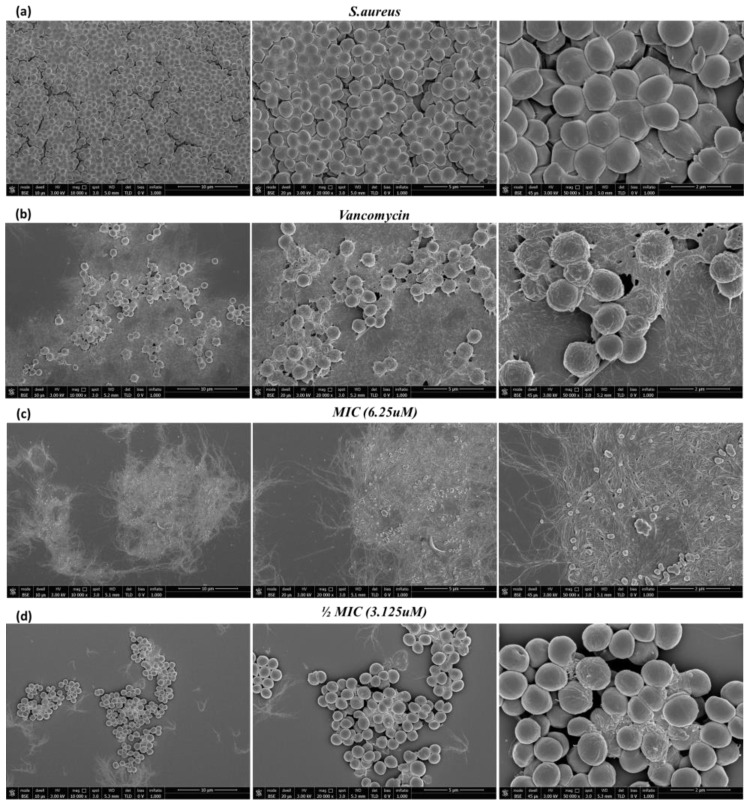
SEM analyses of *S. aureus* cells treated with Hylin-a1. (**a**) Untreated bacteria; (**b**) bacteria treated with vancomycin (6.9 μM) as a positive control; (**c**): bacteria treated with MIC of Hylin-a1 (6.25 µM); (**d**): bacteria treated with ½MIC of Hylin-a1 (3.125 µM). The images were obtained at 10,000×, 20,000×, and 50,000× magnification.

The morphological changes of the bacterial cells were analyzed by treating *S. aureus* cells with Hylin-a1 at concentrations of 2MIC, MIC and ½MIC for 60 min. As shown in Figure 8, the peptide caused the disruption of the bacterial membrane compared to untreated controls. 

In fact, it can be observed that the untreated bacteria were characterized by an intact and regular surface preserving the typical coccoid morphology, which is slightly altered in Figure 8d, i.e., when the bacterium was treated with the peptide at the concentration of ½MIC. 

On the other hand, the peptide at concentrations corresponding to the MIC and higher showed a high inhibitory power by completely destroying and killing the pathogens (Figure 8c). These promising results confirmed that Hylin-a1 exerted both a bacteriostatic action by inhibiting bacterial growth and a bactericidal action by degrading and destroying *S. aureus* only after 1 h of treatment. These data are in accordance with what was recently observed by Park at al. [37], who analyzed Hylin-a1 and its analogs for their ability to block A. baumannii infection. In detail, treatment with 1 MIC Hylin-a1 caused a cell damage of 81.01% and, consequently, bacterial death.

### 2.6. Inflammatory Response

The innate immune system plays a vital role in the host response to any infection [46,47]. In general, when *S. aureus* infects host cell, leukocytes are activated and migrate into the bloodstream, where neutrophils modulate the expression of various molecules (e.g., molecules used in cell adhesion) and cytokines (e.g., interleukins) [48]. 

Here, the inflammatory activity was investigated by treating human keratinocytes with *S. aureus* ATCC (at MOI 0.1) and Hylin-a1 (at the MIC of 6.25 µM) and detecting the expression level of pro-inflammatory cytokines by quantitative real-time PCR (qPCR). 

Three different pro-inflammatory cytokines were evaluated: interleukin 8 (IL-8), interleukin 6 (IL-6) and interleukin 1-beta (IL-1β), which are mainly involved in inflammatory diseases [47,49,50].

**Figure 9:**
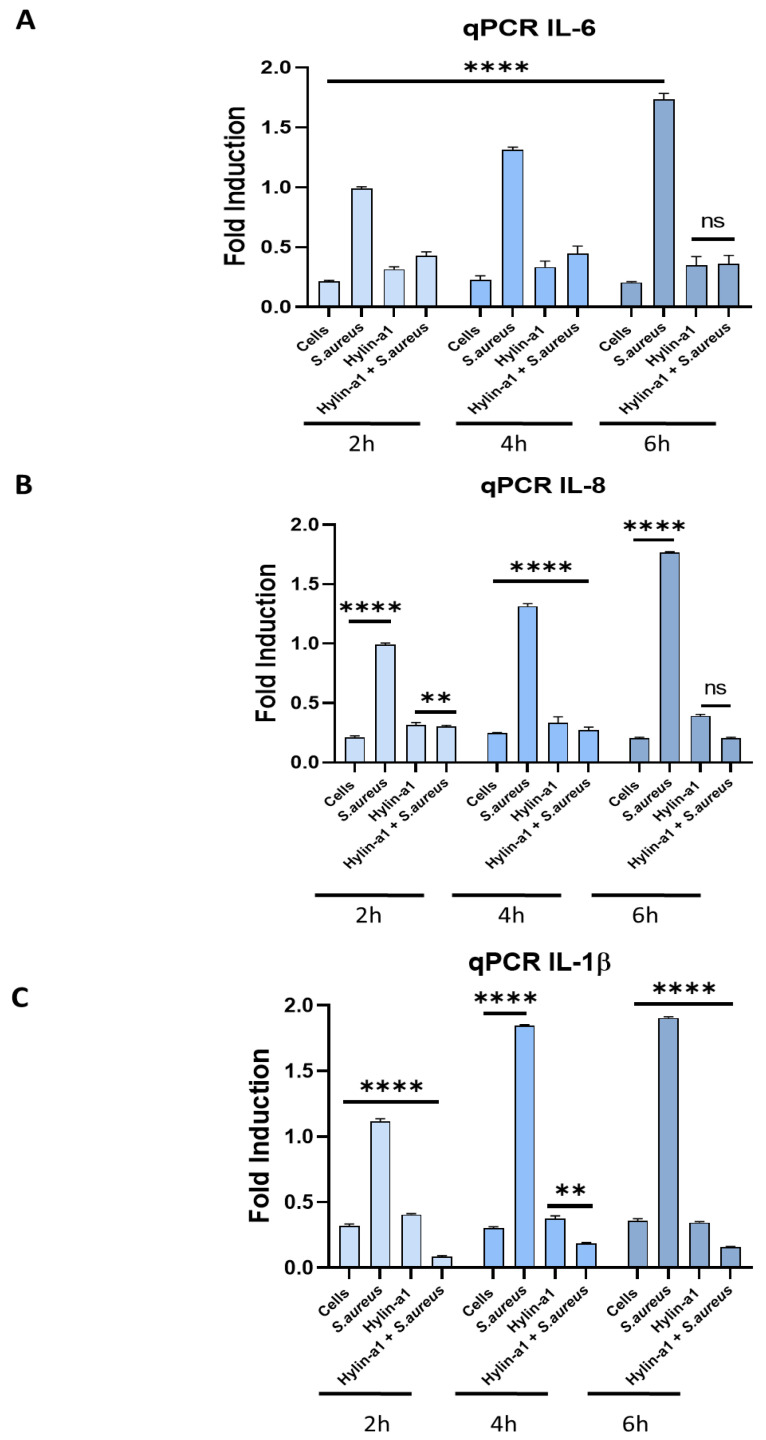
Real-time PCR. Evaluation of pro-inflammatory cytokines. (**A**) Evaluation of IL-6 expression; (**B**) evaluation of IL-8 expression; (**C**) evaluation of IL-1β expression. **** *p* < 0.0001; ns: non-significant.

As reported in Figure 9, all the tested cytokine gene expression levels (IL-8, IL-6, IL-1β) were consistently increased in HaCaT cells after infection with *S. aureus* compared to non-infected cells, and in line with previous results, a remarkable reduction in the expression level of genes was observed when the cells were infected with *S. aureus* and treated with the peptide (Figure 9).

## 3. Materials and Methods

### 3.1. Peptide Synthesis and Characterization

Hylin-a1 (single-letter primary sequence: H-IFGAILPLALGALKNLIK-NH2) was synthesized using oxyma/DIC as coupling agents, as described elsewhere [51,52]. 

High-Performance Liquid Chromatography (HPLC) preparative purification was carried out on a WATERS 2545 preparative system (Waters, Milan, Italy) fitted with a WATERS 2489 UV/Visible detector, applying a linear gradient of CH3CN/0.05% trifluoroacetic acid (TFA) in water, 0.05% TFA from 5 to 70% for 20 min, at a flow rate of 12 mL/min. MS characterization of the peptide was performed using an ESI-TOF-MS Agilent 1290 Infinity LC System coupled to an Agilent 6230 time-of-flight (TOF) LC/MS System (Agilent Technologies, Cernusco sul Naviglio, Italy). The LC Agilent 1290 LC module was coupled with a photodiode array (PDA) detector, a 6230 time-of-flight MS detector, a binary solvent pump degasser, a column heater and an autosampler. LC-MS characterization of the peptide was performed using a C18 Waters xBridge column (3 μm, 4.6 × 5.0 mm), applying a linear gradient of CH3CN/0.05% TFA in water, 0.05% TFA from 5 to 70% for 20 min, at a flow rate of 0.2 mL/min. The yield of the peptide, calculated as ((experimental weight of pure peptide)/(theoretical weight) × 100), with the theoretical weight calculated based on the synthesis scale used, was estimated to be about 70%. The relative purity of the peptide was calculated as the ratio of the peak area of the target peptide to the sum of the areas of all detected peaks from the UV chromatograms at 210.4 nm. The purity was >95%.

### 3.2. Cell and Bacteria Culture

The human keratinocyte cell line HaCaT (Cell Lines Service, CLS-, Eppelheim, Germany) was grown in Dulbecco’s Modified Eagle Medium (DMEM) with 4.5 g/L of glucose (Microtech, Naples, Italy) supplemented with an antibiotic solution 100× (Himedia, Naples, Italy) and 10% Fetal Bovine Serum (FBS, Microtech). In the present study, several strains of Gram-positive bacteria were used, including *S. aureus* (ATCC 6538) and *S. aureus* human clinical isolates (multi-sensitive (MSSA), methicillin-resistant (MRSA), β-lactamase resistant, macrolide-resistant and quinolone-resistant strains). The clinical isolates were isolated from The Microbiology Laboratory of the University of Campania “Luigi Vanvitelli” Hospital, Naples, Italy.

### 3.3. Peptide Cytotoxicity

Hylin-a1 cytotoxicity was evaluated via 3-(4,5-dimethylthiazol-2-yl)-2,5-diphenyltetrazolium bromide (MTT). Several concentrations of the peptide, ranging from 100 to 0.39 μM, were tested. The cells were seeded at 2 × 10^4^ cells/well in 96-well plates (Falcon, NJ, USA) and treated with the peptide for 1 and 24 h. After incubation, 100 μL of MTT solution (5 mg/mL) (Sigma-Aldrich, St. Louis, MO, US) was added to each well, and the plate was incubated at 37 °C/5% CO2 for 3 h. To dissolve the formazan crystals, 100 μL of dimethyl sulfoxide (DMSO, Sigma-Aldrich) was added, and the absorbance was detected by the Sunrise Microplate Absorbance Reader (Tecan, Grodig-Austria) at the wavelength of 570 nm. The data were analyzed, and the percentage of cell viability was reported with respect to the viability of untreated cells (+).

### 3.4. Antibacterial Activity

#### 3.4.1. Determination of Minimum Inhibitory Concentration (MIC) and Minimal Bactericidal Concentration (MBC)

Susceptibility testing was performed following the broth microdilution method reported by the National Committee on Clinical Laboratory Standards (NCCLS), using sterile 96-well plates. The peptide concentrations were selected based on the cytotoxicity revealed by the MTT assay. Hylin-a1 dilutions (50 to 0.39 μM) were prepared in Brain Heart Infusion medium (BHI), adding a volume of 100 μL/well. Then, 50 μL of the standardized bacterial inoculum, corresponding to approximately 5 × 10^5^ colony-forming units (CFU)/mL, was inoculated in each well. The antibacterial activity was expressed as MIC, identifying the lowest concentration of the peptide which completely inhibited bacterial growth after 24 h of incubation at 37 °C. The absorbance was measured by the Tecan instrument at OD600, and the data are expressed as a percentage of inhibition. After MIC determination, 50 μL from all tubes showing no visible bacterial growth, was seeded on BHI agar plates, which were incubated for 24 h at 37 °C. Subsequently, the agar plates were observed, and the MBC was assessed. It was reached when 99.9% of the bacterial population was killed at the lowest concentration of the peptide.

#### 3.4.2. Time-Kill Kinetics Assay

An *S. aureus* inoculum was prepared and incubated at 37 °C overnight. The experiment was conducted in the same condition as the MIC assay, counting the CFU at defined intervals (2,4,6, and 24 h). In addition, 10 μL of serially diluted samples (2MIC, MIC and ½MIC) was spread onto BHI agar plates, and the bacterial colonies were counted after 24 h of incubation at 37 °C.

#### 3.4.3. Invasion Assay

To investigate the role of Hylin-a1 in *S. aureus* invasion, an invasion assay was performed as essentially described elsewhere [53,54]. HaCaT cells were seeded into 24-well plates (1 × 10^5^ cells/well) and grown to ~70–80% confluency. The day after, the cell monolayers were washed with Phosphate-Buffered Saline (PBS, Microtech), and the cells were starved for 2 h with a medium without antibiotics and then treated with Hylin-a1 at different concentrations for 1 h at 37 °C. After a preincubation, the peptide was removed, and the cells were subsequently infected with 1.5 × 10^8^ CFU/mL of *S. aureus*. After 4 h of infection, the cells were washed with PBS for three times, then incubated with gentamicin (Sigma-Aldrich, 100 μg/mL) to kill all extracellular bacteria. After 2 h in the presence of gentamicin, the cells were lysed with a 0.1% Triton-X solution to evaluate the number of intracellular bacteria. Serial dilutions of the cell lysates were performed in 1X PBS, plated on BHI agar, and incubated at 37 °C overnight. Then, the colonies were counted, and the results were expressed by calculating the percentage of bacterial invasion.

### 3.5. Antibiofilm Activity

The antibiofilm activity of the peptide was evaluated against *S. aureus* ATCC by analyzing different steps of biofilm formation and development. Three tests were performed in vitro to evaluate the initial attachment, formation, degradation and penetration of the biofilm.

#### 3.5.1. Initial Attachment Assay

An overnight-grown bacterial inoculum was diluted to a concentration of ~8 × 10^7^ CFU/mL in BHI supplemented with 1% (*v*/*v*) glucose. Different non-cytotoxic concentrations of the peptide were tested in the range of 100 to 0.78 µM. For this assay, 200µL of the bacterial suspension and Hylin-a1 was added to each well of 96-well plates. After 1 h of incubation, non-adherent plankton cells were removed by washing with 1× PBS. MTT (3-(4,5-dimethylthiazol-2-yl)-2,5-diphenyltetrazolium bromide) was added, and the plates were incubated for 2 h at 37 °C. The percentage of cell adhesion was evaluated by measuring the absorbance at 570 nm and normalizing the value of each well with that of the untreated control.

#### 3.5.2. Biofilm Inhibition Assay

The inhibition of biofilm formation was evaluated by performing a colorimetric assay using crystal violet (CV). The bacterial strain *S. aureus* was cultured overnight in BHI, and the absorbance at OD600 was adjusted to 0.1 in medium with 1% glucose. For this assay, 200 μL of bacterial suspension and peptide (at different concentrations) was seeded in a 96-well plate and incubated O/N at 37 °C. Then, the plates were washed with 1X PBS, dried and stained with 0.1% CV for 30 min at room temperature (RT). Finally, 200 µL of 99% ethanol was used to resolubilize the biofilm, and the absorbance was measured at 570 nm. The percent inhibition was calculated by normalizing each well with respect to the untreated control.

#### 3.5.3. Biofilm Degradation Assay

The degradation activity of the biofilm was evaluated by the crystal violet colorimetric assay. The bacterial cells were inoculated in BHI supplemented with 1% glucose at 37 °C O/N. 

As for the inhibition assay, previously described, the inoculum was adjusted to 0.1 OD600, and 100 µL of the bacterial suspension was seeded in a 96-well plate and incubated at 37 °C for 24 h. Following biofilm formation, 100 µL of the peptide at different concentrations was seeded in a 96-well plate and incubated at 37 °C O/N. Then, 100 µL of CV at 0.1% (*v*/*v*) was inoculated into each well, and the plate was incubated for 40 min at RT. Finally, CV was solubilized with 95% ethanol for 20 min, and the biofilm degradation rate was calculated by measuring the absorbance at 570 nm and normalizing each value with respect to that of the untreated control.

### 3.6. FACScan Analysis

To evaluate the cell membrane integrity following the peptide treatment, a flow cytometry test was performed [55]. Log-phase *S. aureus* bacterial cells were harvested by centrifuge and treated with the peptide at concentrations of 2MIC, MIC and ½MIC. The samples were incubated for 30 min at 28 °C with constant shaking (140 rpm). Subsequently, 10 µg/mL of propidium iodide (PI) was added, and the samples were incubated for 30 min at 4 °C. After incubation, washings with saline (1X PBS) were performed to remove the excess unbound dye, and flow cytometry analysis by BD FACSCanto II (Franklin Lakes, NJ, USA) was conducted. *S. aureus* cells incubated with PI without the peptide were used as a negative control.

### 3.7. Scanning Electron Microscopy (SEM)

Scanning electron microscopy analyses were performed to evaluate the morphological changes induced by Hylin-a1.

*S. aureus* ATCC was grown in BHI broth to log phase and collected by centrifugation (1000× *g* for 10 min). The bacterial pellets were adjusted to OD600 of 0.2 with PBS (10 mM). Different concentrations of the peptide were incubated with the bacterial suspension for 60 min at 37 °C. Subsequently, the bacterial cells were centrifuged, washed with PBS and fixed in 2.5% (*v*/*v*) glutaraldehyde O/N at 4 °C. Vancomycin and untreated bacteria were used as positive and negative controls, respectively. 

Finally, the bacteria were washed with PBS and dehydrated with increasing concentrations of ethanol (50, 70, 90, 10% *v*/*v*), each step for 15 min. Images were acquired by SEM using the ZEISS Supra 40 instrument (EHT = 5.00 kV, WD = 22 mm, detector in the objective) (Berlin, Germany) [55].

### 3.8. Quantitative Real-Time PCR

HaCaT cells were seeded in 12-well plates at 2 × 10^5^ cells/well. The next day, the cell monolayer was infected with *S. aureus* (1 × 10^8^ CFU/mL, OD 0.1) and treated with Hylin-a1 at the MIC of 6.25 μM. Evaluations were performed at several times of incubation (2, 4 and 6 h). Subsequently, RNA was extracted with the TRIzol reagent (Thermo Fisher Scientific, USA) and quantified by assessing the absorbance by Nanodrop (NanoDrop 2000, Thermo Fisher Scientific). The All-In-One RT MasterMix 5X (Applied Biological Materials, Canada) was used to reverse-transcribe RNA into cDNA, and 2 µL of cDNA was amplified by real-time PCR. The expression of the following genes was assessed: interleukin (IL)-1β, IL-6 and IL-8. The target threshold cycle (Ct) values were normalized to glyceraldehyde-3-phosphate dehydrogenase (GAPDH), which was used as a housekeeping gene. The mRNA levels were expressed using the 2^−ΔΔCt^ method. The primer sequences for real-time PCR are shown in Table 3 and were acquired from Eurofins Genomics (Plantside Dr Louisville, KY, USA).

### 3.9. Statistic Analysis

All tests were performed in triplicate and re expressed as mean ± standard deviation (SD) calculated by GraphPad Prism (version 8.0.1). One-way ANOVA followed by the Dunnett’s multiple comparisons test was applied; a value of *p*  < 0.001 was considered significant.

## 4. Conclusions

In summary, to cope with the emergency of antibiotic resistance, we evaluated the antibacterial activity of Hylin-a1. It is known that the cutaneous secretion of amphibians represents a resource of biologically active peptides with strong antimicrobial effect. Through a series of molecular and cellular studies conducted in vitro, the peptide showed remarkable antibacterial activity with low toxicity towards eukaryotic cells at the active concentrations. In detail, here we showed that the inhibition of bacterial invasion using Hylin-a1 diminished the level of cytokine observed in response to infection with *S. aureus*. The activation of cytokine production by invasive bacteria is transient, even when the bacterial stimulus persists in the intracellular environment; therefore, treatment with Hylin-a1 reduced bacteria invasion and the related cytokine cascade. 

These data suggest that Hylin-a1 may be a good candidate for developing novel antimicrobial agents to treat antibiotic-resistant infections.

## Figures and Tables

**Table 1:** Summary of MICs and MBCs evaluated for Hylin-a1 against different *S. aureus* strains.

Bacteria	MIC (μM)	MBC (μM)
*S.aureus* ATCC 6538	6.25	6.25
Multi-sensitive *S.aureus*	6.25	-
Macrolide-resistant *S.aureus*	6.25	-
Methicillin-resistant *S.aureus*	3.125	-
Quinolone-resistant *S.aureus*	6.25	-
β-lactamase resistant *S.aureus*	3.125	-

**Table 2:** Flow cytometry analysis. Median fluorescence values for each compound tested.

Name	Median Fluorescence
*S. aureus* ATCC 6538	61
Hylin-a1 (1/2 MIC)	64
Hylin-a1 (2 MIC)	192
Hylin-a1 (MIC)	166
Vancomycin	181

**Table 3:** Primers for the inflammation analysis.

Gene	Forward Sequence	Reverse Sequence
IL-1β	GCATCCAGCTACGAATCTCC	CCAACATTCAGCACAGGACTC
IL-6	AATAACCACCCCTGACCCAAC	ACATTTGCCGAAGAGCCCT
IL-8	AAACCACCGGAAGGAACCAT	CCTTCACACAGAGCTGCAGAAA
GAPDH	CCTTTCATTGAGCTCCAT	CGTACATGGGAGCGTC

## Data Availability

The data presented in this study are available on request from the corresponding author. Authors can confirm that all relevant data are included in the article.
